# Supplementary material for: Observation of continuum Landau modes in non-Hermitian electric circuits
Source: Nat Commun. 2024 Feb 27;15:1798. doi: 10.1038/s41467-024-46122-0 (PMC10899205; doi:10.1038/s41467-024-46122-0)
Supplement: Supplementary file 2 — Supplementary Information [file 41467_2024_46122_MOESM2_ESM.pdf]

Supplementary Information for "Observation of continuum Landau modes in non-Hermitian electric circuits"

Xuewei Zhang[1,2*], Chaohua Wu[1*], Mou Yan[1,3], Ni Liu[4], Ziyu Wang[5†], Gang Chen[1,2†]

[1]School of Physics and Microelectronics, Key Laboratory of Materials Physics of Ministry of Education, Zhengzhou University, Zhengzhou 450001, China

[2]State Key Laboratory of Quantum Optics and Quantum Optics Devices, Institute of Laser spectroscopy, Shanxi University, Taiyuan 030006, China

[3]Institute of Quantum Materials and Physics, Henan Academy of Sciences, Zhengzhou 450046, China

[4]Institute of Theoretical Physics, Shanxi University, Taiyuan 030006, China

[5]The Institute of Technological Sciences, Wuhan University, Wuhan 430072, China

*These authors contributed equally to this work.

†Corresponding author. Email: zywang@whu.edu.cn; chengang971@163.com

This Supplementary Information includes:

Supplementary Note 1: Effective circuit model (Supplementary Figure 1)

Supplementary Note 2: Continuum Landau modes (Supplementary Figure 2)

Supplementary Note 3: The role of driving frequency on the admittance eigenvalues and eigenstates (Supplementary Figures 3 and 4)

Supplementary Note 4: Complex eigenfrequency spectrum (Supplementary Figure 5)

Supplementary Note 5: Measurement methods in experiment (Supplementary Figures 6 and 7)

Supplementary Note 6: The effect of circuit components errors on continuum Landau modes (Supplementary Figure 8)

Supplementary Note 7: Experimental results of the frequency-controlled admittance spectrum and eigenstates (Supplementary Figure 9)

Supplementary Note 8: Continuum Landau modes in one-dimensional circuit lattices (Supplementary Figures 10, 11, and 12)

Supplementary Note 1: Effective circuit model

We employ a 2D electric circuit network that contains non-reciprocal hopping and linear complex on-site potential to realize the continuum Landau modes. The scheme of a part of designed electric circuit is shown in Supplementary Figure 1. Each node is connected to two adjacent nodes through capacitors C_1 along the x direction and to

two adjacent nodes through impedance converter with current inversion (INIC) of capacitance $\pm C_2$ along the y direction. Moreover, the nodes are grounded by inductors L_0 as well as position-dependent capacitors nC_0 and resistors R_0/m. According to the Kirchhoff's law, the response of the circuit at frequency ω is described by $\boldsymbol{I}(\omega) = J(\omega)\boldsymbol{V}(\omega)$, where $J(\omega)$ is the admittance matrix or circuit Laplacian, and the vector components of $\boldsymbol{I}$ and $\boldsymbol{V}$ correspond to the input currents and voltages at the nodes or sites in the circuit, respectively. The current-voltage relation of the node (m,n) is governed by

$$\begin{aligned} I_{mn} = {} & i\omega C_1\left[\left(V_{mn} - V_{m+1,n}\right) + \left(V_{mn} - V_{m-1,n}\right)\right] + i\omega C_2\left(V_{mn} - V_{m,n+1}\right) \\ & -i\omega C_2\left(V_{mn} - V_{m,n-1}\right) + \left(i\omega n C_0 + \frac{1}{i\omega L_0} + \frac{m}{R_0}\right)V_{mn}. \end{aligned} \tag{1}$$

Then the admittance matrix can be obtained as

$$\begin{aligned} \frac{J(\omega)}{i\omega} = {} & \sum_{m,n}\left[t_x(|m,n\rangle\langle m+1,n| + \mathrm{H.c.}) + t_y(|m,n+1\rangle\langle m,n| - \mathrm{H.c.})\right. \\ & \left. +\left(nB_y - imB_x + \epsilon_0\right)|m,n\rangle\langle m,n|\right], \end{aligned} \tag{2}$$

where $t_x = -C_1$, $t_y = -C_2$, $B_y = C_0$, $B_x = 1/(\omega R_0)$, and $\epsilon_0 = C_1 - 1/(\omega^2 L_0)$ The admittance matrix J plays the role of the Hamiltonian matrix. Notably, the non-reciprocal hoppings are implemented and controlled through the INIC, while the linear real and imaginary potentials are respectively realized by the position-dependent capacitors nC_0 and resistors R_0/m.

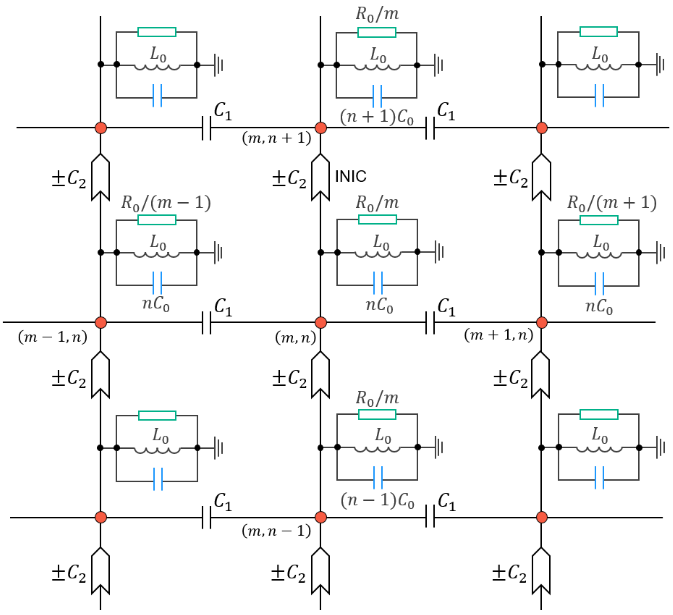

Supplementary Figure 1. **Schematic diagram of a part of the designed circuit.** The adjacent nodes (red dots) are connected through capacitors C_1 along the x direction and connected through INIC with $\pm C_2$ along the y direction. Each node is grounded by an inductor L_0 as well as a position-dependent capacitor nC_0 (blue capacitors) and a resistor R_0/m (green resistors).

Supplementary Note 2: Continuum Landau modes

In order to clarify the phenomenon of continuum Landau modes (CLMs), we consider the continuum limit of the lattice model in Supplementary Figure 1. By taking $|\psi_{\mathbf{k}}\rangle = \sum_{m,n} e^{(ik_x m+ik_y n)}\Psi_{mn}\,|m,n\rangle$, Supplementary Equation 2 can be rewritten as

$$\frac{J(\omega)}{i\omega}|\psi_{\mathbf{k}}\rangle = \sum_{m,n}\Big\{(nB_y - imB_x + \epsilon_0)\Psi_{mn}e^{(ik_x m+ik_y n)} + t_x\Big[e^{[ik_x(m+1)+ik_y n]}\Psi_{m+1,n} + e^{[ik_x(m-1)+ik_y n]}\Psi_{m-1,n}\Big] + t_y\Big[e^{[ik_x m+ik_y(n-1)]}\Psi_{m,n-1} - e^{[ik_x m+ik_y(n+1)]}\Psi_{m,n+1}\Big]\Big\}|m,n\rangle. \quad (3)$$

Applying the slowly-varying envelope approximation $\Psi_{m\pm1,n} \approx \Psi_{mn} \pm \partial_x\Psi|_{mn}$ and $\Psi_{m,n\pm1} \approx \Psi_{mn} \pm \partial_y\Psi|_{mn}$ by ignoring the higher-order terms, Supplementary Equation 3 becomes

$$\frac{J(\omega)}{i\omega}|\psi_{\mathbf{k}}\rangle = \sum_{m,n}\Big[(nB_y - imB_x + \epsilon_0)\Psi_{mn} + i2t_x\sin k_x\frac{\partial\Psi}{\partial x} + 2t_x\cos k_x\Psi_{mn} - i2t_y\sin k_y\Psi_{mn} - 2t_y\cos k_y\frac{\partial\Psi}{\partial y}\Big]e^{(ik_x m+ik_y n)}|m,n\rangle. \quad (4)$$

From Supplementary Equation 4, the slowly-varying envelope satisfies $J_{\mathbf{k}}/(i\omega)\Psi_{mn} = j/(i\omega)\Psi_{mn}$ with

$$J_{\mathbf{k}}/(i\omega) = \mathcal{E}_{\mathbf{k}}^0 - (-i\mu_{\mathbf{k}}\partial_x - B_y y) + i(-i\nu_{\mathbf{k}}\partial_y - B_x x), \quad (5)$$

where $\mathcal{E}_{\mathbf{k}}^0 = 2t_x\cos k_x - i2t_y\sin k_y + \epsilon_0$, $\mu_{\mathbf{k}} = 2t_x\sin k_x$, and $\nu_{\mathbf{k}} = -2t_y\cos k_y$.

It should be noticed that $J_{\mathbf{k}}/(i\omega)$ is equivalent to the non-Hermitian Dirac Hamiltonian with first-order imaginary momentum under a uniform magnetic field. In this perspective, the pseudovector potential is given by $\mathbf{A} = (-B_y y, B_x x)$, which corresponds to a uniform pseudomagnetic field $\mathbf{B} = \nabla\times\mathbf{A} = (B_x + B_y)\hat{z}$. It has been demonstrated that such non-Hermitian Dirac Hamiltonian features CLMs. To clarify this point, we first focus on the 2D Hermitian Dirac Hamiltonian described by

$$\mathcal{H}_{\mathbf{k}} = \begin{bmatrix} 0 & J_{\mathbf{k}}/(i\omega) \\ [J^*_{\mathbf{k}}/(-i\omega)] & 0 \end{bmatrix}. \tag{6}$$

It is known that the corresponding pseudomagnetic field $\mathbf{B}$ leads to discrete Landau levels in the spatial spectrum. For the zeroth ($E = 0$) Landau levels, the two-component wavefunction $\psi_0 = (\psi_1, \psi_2)^{\mathrm{T}}$ satisfies

$$J_{\mathbf{k}}/(i\omega)\psi_2 = 0, \tag{7a}$$

$$[J^*_{\mathbf{k}}/(-i\omega)]\psi_1 = 0. \tag{7b}$$

Through a straightforward calculation, we have

$$\psi_1 = 0, \quad \psi_2 = Ce^{-\eta_x(x-x_0)^2}e^{-\eta_y(y-y_0)^2}e^{i\mathbf{q}\cdot\mathbf{r}}, \tag{8}$$

where $\eta_x = -B_x/2\mu_{\mathbf{k}}$, $\eta_y = B_y/2\nu_{\mathbf{k}}$, C is the normalized coefficient, and the center position

$$\mathbf{r}_0(\mathbf{k},\mathbf{q}) = (\mathrm{Im}(\mathcal{E}^0_{\mathbf{k+q}})/B_x, -\mathrm{Re}(\mathcal{E}^0_{\mathbf{k+q}})/B_y) + O(|\mathbf{q}|^2), \tag{9}$$

with $\mathbf{q} = (q_x, q_y)$ being an arbitrary wavevector. Evidently, the zeroth Landau level modes are Gaussian wavepackets for $B_x/\mu_{\mathbf{k}} < 0$ and $B_y/\nu_{\mathbf{k}} > 0$.

For the non-Hermitian Dirac Hamiltonian described by $J_{\mathbf{k}}/(i\omega)$, the eigenequation reads

$$J_{\mathbf{k}}/(i\omega)\psi_{\mathbf{k}} = j/(i\omega)\psi_{\mathbf{k}}. \tag{10}$$

We can see that Supplementary Equation 10 is equivalent to Supplementary Equation 7a with displacement $\mathbf{r} \to \mathbf{r} + \left[\mathrm{Im}\left(\frac{j}{i\omega}\right)/B_x, \mathrm{Re}\left(\frac{j}{i\omega}\right)/B_y\right]$. Consequently, the eigenstates of Supplementary Equation 10 share the same set of gaussian wavepackets of the zeroth Landau level modes of $\mathcal{H}_{\mathbf{k}}$ but with a displaced center position related to $j/(i\omega)$ i.e.,

$$\mathbf{r}_0(j,\mathbf{k},\mathbf{q}) = \begin{pmatrix} x_0 \\ y_0 \end{pmatrix} = \begin{pmatrix} -\mathrm{Im}\big(j/(i\omega) - \mathcal{E}^0_{\mathbf{k+q}}\big)/B_x \\ \mathrm{Re}\big(j/(i\omega) - \mathcal{E}^0_{\mathbf{k+q}}\big)/B_y \end{pmatrix} + O(|\mathbf{q}|^2). \tag{11}$$

Note that the solutions ψ_0 and ψ are limited in the regime $|\mathbf{q}| \ll 1$ due to the slowly-varying envelope approximation of Ψ_{mn}. Supplementary Equation 11 indicates that the eigenstate $\psi_{\mathbf{k}}$ with a given eigenvalues $j/(i\omega)$ can map to the zeroth Landau level mode of $\mathcal{H}_{\mathbf{k}}$, whose gauge is determined by $j/(i\omega)$. Since the gauge can continuously varied, the eigenvalues $j/(i\omega)$ are uncountable. Moreover, Supplementary Equation 11 suggests that the localized centers of the CLMs are linear dependent on the complex energies.

To verify the behavior of the CLMs in the circuit simulator, we calculate the

admittance spectrum and eigenstates for the circuit Laplacian in Supplementary Equation 2, as shown in Supplementary Figures 2**a** and 2**b**. We can see that the complex eigenvalues form a band over a finite area in the complex energy plane. Moreover, the position expectation values $\langle x \rangle$ and $\langle y \rangle$ are proportional to $\mathrm{Re}[j/(i\omega)]$ and $\mathrm{Im}[j/(i\omega)]$, respectively. The corresponding eigenstates are localized with Gaussian envelops [see Supplementary Figure 2**c**]. These results are in good agreement with the theoretical predictions.

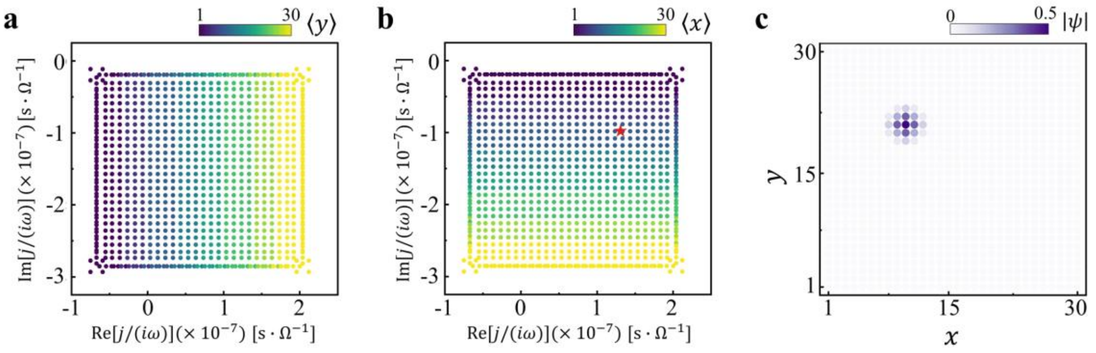

Supplementary Figure 2. **Admittance spectra of the circuit Laplacian.** (**a**, **b**) Calculated admittance spectra of the circuit Laplacian Supplementary Equation 2 for the frequency $f = 162$ kHz. The colors in **a** and **b** denote the position expectation values $\langle x \rangle$ (**a**) and $\langle y \rangle$ (**b**), respectively. **c** The distribution of the eigenstate marked by star in **b**. Here, we take $N_x = N_y = 30$, $C_0 = 10$ nF, $L_0 = 10$ μH, $R_0 = 100\ \Omega$, $C_1 = 10$ nF, and $C_2 = 10$ nF.

Supplementary Note 3: The role of the a.c. frequency on the admittance eigenvalues and eigenstates

The frequency is a tuning parameter incorporated in the circuit Laplacian (Supplementary Equation 2) with the frequency-dependent coefficient ϵ_0 and pseudomagnetic field B_x. In the following, we illustrate the effects of these two frequency-dependent parameters on the admittance spectrum and eigenstates, respectively.

The frequency modifies the admittance spectrum. We first note that, from the circuit Laplacian $J(\omega)/(i\omega)$, the diagonal components ϵ_0 and B_x induce the shift of

the admittance spectrum along the directions of $\mathrm{Re}[j/(i\omega)]$ and $\mathrm{Im}[j/(i\omega)]$, respectively. Due to the existence of the site index m before B_x, the bandwidth of $\mathrm{Im}[j/(i\omega)]$ may vary with the frequency. On the other hand, by requiring the center position $\mathbf{r}_0$ of the CLMs [Supplementary Equation 11] to lie in the lattice, we obtain the boundaries of the admittance spectrum as $B_y + \epsilon_0 - 2|t_x| \leq \mathrm{Re}[j/(i\omega)] \leq B_y N_x + \epsilon_0 + 2|t_x|$ and $-B_x N_x - 2|t_y| \leq \mathrm{Im}[j/(i\omega)] \leq -B_x + 2|t_y|$. It can be seen that the coefficient ϵ_0 causes the shift of $\mathrm{Re}[j/(i\omega)]$, while B_x not only gives rise to the shift of $\mathrm{Im}[j/(i\omega)]$, but also influences its bandwidth.

Supplementary Figures 3**a** and 3**b** show the complex admittance spectra for the frequencies $f = 162$ kHz (**a**) and $f = 200$ kHz (**b**), respectively. Obviously, the size and distributions of the admittance spectrum change with the frequency. We further plot the calculated results of $\mathrm{Re}[j/(i\omega)]$ and $\mathrm{Im}[j/(i\omega)]$ versus the frequency f, as shown respectively in Supplementary Figures 3**c** and 3**d**. We can find that $\mathrm{Re}[j/(i\omega)]$ varies with the frequency f and its bandwidth stays almost the same, as expected. While the bandwidth of $\mathrm{Im}[j/(i\omega)]$ reduces with the increasing of the frequency.

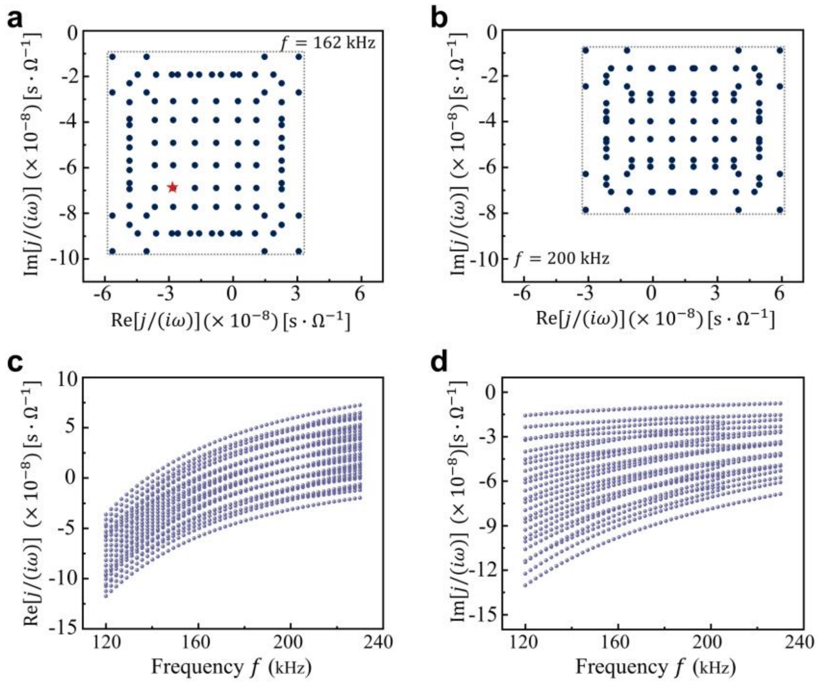

Supplementary Figure 3. **The role of the frequency on the admittance eigenvalues.** (**a**, **b**) Calculated admittance spectra of the circuit Laplacian [Eq. (1) in the main text]

for the different frequencies $f = 162$ kHz (**a**) and 200 kHz (**b**). The color of each point indicates the participation ratio of the corresponding eigenstate. (**c**, **d)** Calculated results of $\mathrm{Re}[j/(i\omega)]$ (**c**) and $\mathrm{Im}[j/(i\omega)]$ (**d**) versus the frequency f. The parameters are the same as those in Fig. 1 of the main text.

The frequency also affects the localization of the admittance eigenstates as well as the slope of the linearity between the CLM's center position and complex eigenvalues. On one hand, from the wavefunction ψ_0 of the CLM, the localization of the Gaussian envelope is characterized by $\eta_x = -B_x(\omega)/(2\mu_{\mathbf{k}})$ and $\eta_y = B_y/(2\nu_{\mathbf{k}})$. That is to say, the frequency only affect the localization of the CLMs in the x direction. Supplementary Figures 4**a** and 4**b** show the calculated and simulated amplitude distribution along lines passing through the center of one eigenstate (marked by star in Supplementary Figure 3**a**) for the different frequencies, respectively. These calculated and simulated results agree well with the theoretical predictions. However, the effect ($\sim 1/\omega$) is very weak and is thus hard to be observed in experiment.

On the other hand, according to Supplementary Equation 11, the linear relationships between complex admittance eigenvalues and the CLM's center position are given by $\mathrm{Re}[j/(i\omega)] = B_y y_0 + \epsilon_0$ and $\mathrm{Im}[j/(i\omega)] = -B_x x_0$. We can see that the frequency only introduces a shift constant in the linearity between $\mathrm{Re}[j/(i\omega)]$ and y_0, while it can tune the slope of the linearity between $\mathrm{Im}[j/(i\omega)]$ and x_0. In Supplementary Figures 4**c** and 4**d**, we show the real and imaginary parts of the admittance eigenvalues, $\mathrm{Re}[j/(i\omega)]$ and $\mathrm{Im}[j/(i\omega)]$, as functions of the eigenstate's position expectation values $\langle y \rangle$ and $\langle x \rangle$ for the different frequencies, respectively. The numerical results are consistent with the theoretical analysis.

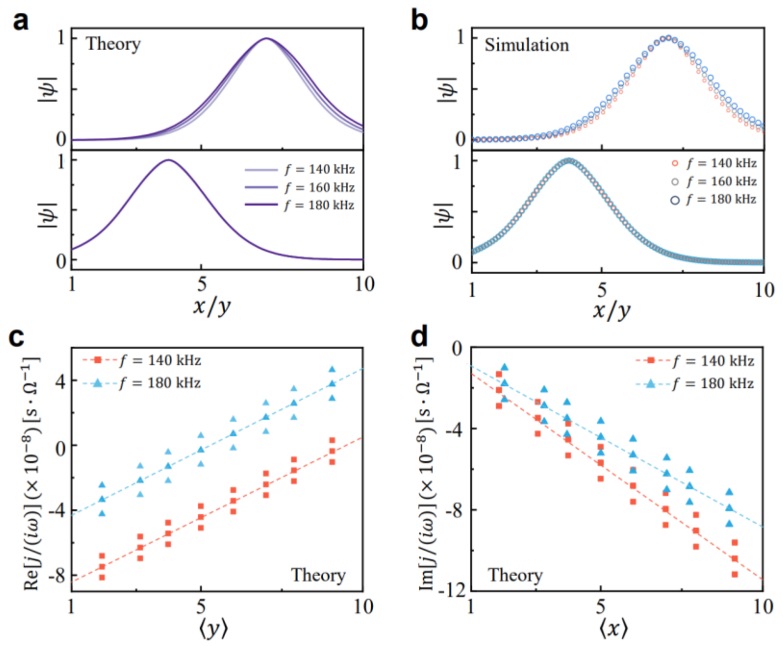

Supplementary Figure 4. **The role of the frequency on the admittance eigenstates.** (**a**, **b**) Calculated (**a**) and simulated (**b**) amplitude distribution along lines passing through the center of one eigenstate (marked by star in Supplementary Figure 3**a**) for the different frequencies $f = 140$, 160, and 180 kHz. The upper and lower panels indicate the results along the x and y directions, respectively. **c** Calculated results of $\mathrm{Re}[j/(i\omega)]$ versus the eigenstate's position expectation value $\langle y \rangle$ for the different frequencies $f = 140$ and 180 kHz. **d** Calculated results of $\mathrm{Im}[j/(i\omega)]$ versus the eigenstate's position expectation value $\langle x \rangle$ for the different frequencies $f = 140$ and 180 kHz. The dashed lines in **c** and **d** denote the theoretical central trend lines ($\mathcal{E}^0_{\mathbf{k}+\mathbf{q}} \to 0$) obtained from Supplementary Equation 11. The parameters are the same as those in Fig. 1 of the main text.

Supplementary Note 4: Complex eigenfrequency spectrum

As an external parameter, the frequency in the circuit Laplacian can be tuned at will. For each frequency, the eigenvalues of the admittance matrix fill the complex energy plane if the lattice is infinite, i.e., they form a continuum. The roots of the admittance spectrum $j(\omega) = 0$ corresponds to the complex eigenfrequency spectrum of the system. As shown in Supplementary Figure 5, this complex eigenfrequency

spectrum has the same number of the eigenstates as the complex admittance spectrum, and can thus form a continuum filling the complex frequency space. When the complex admittance or eigenfrequency spectra form a continuum, the voltage response is continuous, i.e., any frequency can excite the corresponding eigenmode of the circuit.

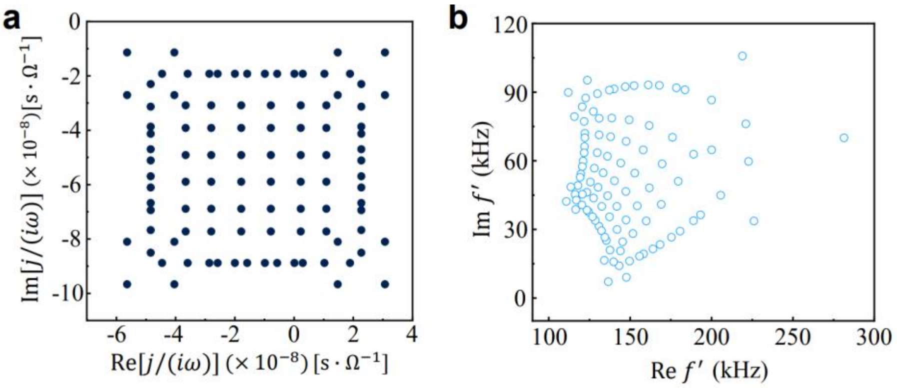

Supplementary Figure 5. **Admittance and complex eigenfrequency spectra. a** Calculated admittance spectrum of the circuit Laplacian in Supplementary Equation 2 fed by the frequency $f = 162$ kHz. The color of each point indicates the participation ratio of the corresponding eigenstate. **b** Complex eigenfrequency spectrum (f') close to the frequency $f = 162$ kHz. The parameters are the same as those in Fig. 1 of the main text.

Supplementary Note 5: Measurement methods in experiment

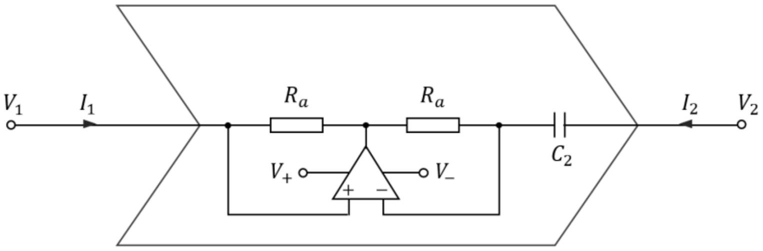

Supplementary Figure 6. **Impedance converter.** Circuit diagram of the INIC used to realize the non-reciprocal hopping between two nodes, which consists of an operational amplifier, a resistor R_a and a capacitor C_2.

As mentioned previously, the key element in our circuit is the INIC, which incorporates an operational amplifier (OpAmp) in a negative feedback configuration, as shown in Supplementary Figure 6. Assuming that the negative potential is ideally equal to the positive input potential V_1, the input currents I_1 and output I_2 depending on the node voltages V_1 and V_2 can be calculated as

$$I_1 = I_2 = \frac{(V_1 - V_0)}{R_a}, \tag{12a}$$

$$I_2 = i\omega C_2 (V_2 - V_1), \tag{12b}$$

Translating these results to the Laplacian form leads to the node voltage equation

$$\begin{pmatrix} I_1 \\ I_2 \end{pmatrix} = i\omega C_2 \begin{pmatrix} -1 & 1 \\ -1 & 1 \end{pmatrix} \begin{pmatrix} V_1 \\ V_2 \end{pmatrix}. \tag{13}$$

It is shown that the INIC implements a positive capacitance C_2 in one direction and a negative capacitance $-C_2$ in the opposite direction, inducing a non-reciprocal connection between two nodes. In the experiment, we used the unity-gain stable operational amplifier model LT1363.

The direct observables in circuit experiment are the node voltages and input currents. In the Laplacian formalism of circuit arrays, the admittance eigenvalues and eigenstates can be reconstructed from voltage and current measurements. For a circuit of N nodes labeled by the index $j = 1, \cdots, N$, the voltage response to an external current excitation at node i is given by

$$\boldsymbol{V} = \begin{pmatrix} G_{1,i} I_i \\ \vdots \\ G_{N,i} I_i \end{pmatrix} = \begin{pmatrix} G_{1,1} & \cdots & G_{1,N} \\ \vdots & \ddots & \vdots \\ G_{N,1} & \cdots & G_{N,N} \end{pmatrix} \begin{pmatrix} 0 \\ \vdots \\ I_i \\ \vdots \\ 0 \end{pmatrix}, \tag{14}$$

where $G_{j,i} = V_j^{(i)} / I_i$. By repeating this procedure for all input nodes (N times), the complete Green's function G is accessible, which is the inverse of the circuit Laplacian J. Therefore, we can obtain the eigenvalues and eigenstates of the circuit Laplacian by diagonalizing G.

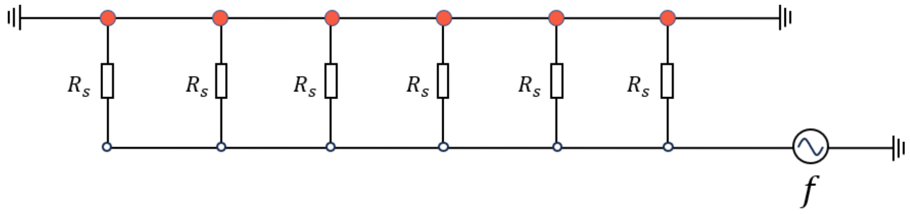

Supplementary Figure 7. Sketch of the voltage response through full-field excitation for the 1D circuit chain.

The linear relationship between the admittance spectrum and the localized position of its eigenstates [Supplementary Figure 2] manifests itself as a novel voltage response. To demonstrate this feature in experiment, we measure the steady-state voltage profile as a function of frequency by full-field excitation. As displayed in Supplementary Figure 7, multichannel a.c. current feeds generated from a voltage source are injected into all nodes through a shunt resistance ($R_s = 50\ \Omega$) separately. Then we measure the voltage distribution on each node at different driving frequency.

Supplementary Note 6: The effect of circuit components errors on continuum Landau modes.

In Fig. 1**c** or Figs. 2**b** and 2**c** of Reference (37), the clean circuit Laplacian/Hamiltonian is considered, and the admittance/energy spectra are thus highly symmetric. In the real experiments, the errors of the electronic components usually exist. For example, in our experiment with the high-precision electronic components, these errors are about $\pm 1\%$. In order to investigate the effects induced by the errors, we use the LTspice software to simulate the admittance spectra and the eigenstate's energy against the position expected values by introducing $\pm 1\%$ (**a**, **b**) and $\pm 5\%$ (**c**, **d**) disorders to all the circuit components. As shown in Supplementary Figure 8**a**. the admittance spectrum turns out to be cluttered, even if the high-precision electronic components with the errors of $\pm 1\%$ are introduced. However, the key features of the CLMs, the localization of the eigenstates and the linear relationship between the eigenstates' center position and eigenvalues, still exist (Supplementary Figure 8**b**). When the errors increase ($\pm 5\%$), the similar properties of the admittance spectra and

the linear relationship between the eigenstates' center position and eigenvalues are found (Supplementary Figures 8**c** and 8**d**), which means that the features of the CLMs are robust to the errors of the electronic components.

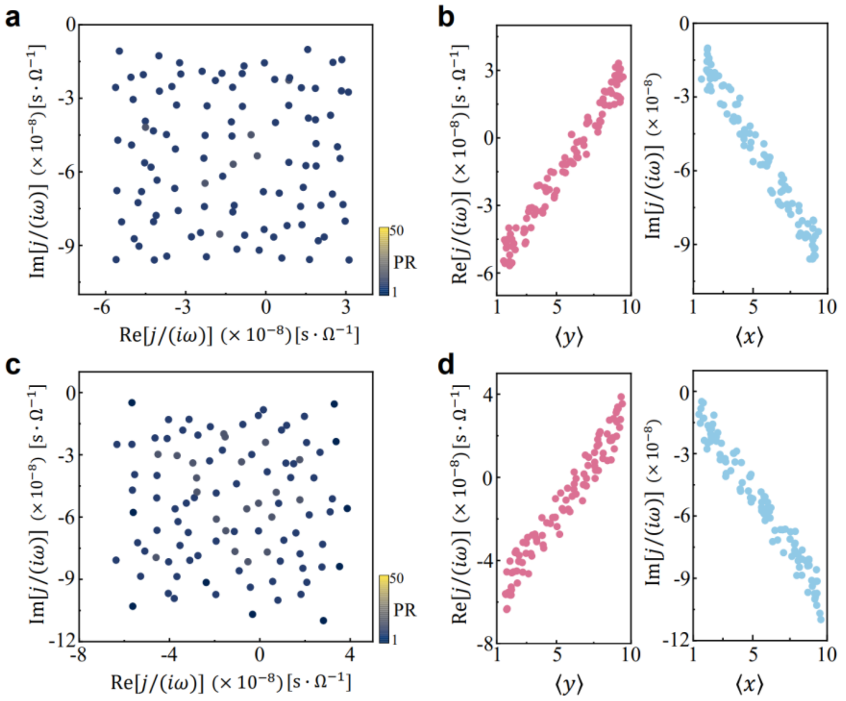

Supplementary Figure 8. **Admittance spectra with component errors. a** Simulated admittance spectrum of the circuit Laplacian by considering the error of $\pm 1\%$ for all circuit components. The color of each point indicates the participation ratio of the corresponding eigenstate. **b** Simulated results of $\mathrm{Re}[j/(i\omega)]$ (Left panel) and $\mathrm{Im}[j/(i\omega)]$ (Right panel) versus the expectation values of the eigenstate's position, $\langle y \rangle$ and $\langle x \rangle$, respectively. (**c**, **d**) The simulated results corresponding to **a** and **b** with the error of $\pm 5\%$ for all circuit components. The other parameters are the same as those in Fig. 2 of the main text.

Supplementary Note 7: Experimental observations of the frequency-controlled admittance spectrum and eigenstates.

As discussed in Supplementary Note 3, the frequency-dependent coefficient ϵ_0 causes the shift of $\mathrm{Re}[j/(i\omega)]$, while the frequency-dependent pseudomagnetic field B_x not only gives rise to the shift of $\mathrm{Im}[j/(i\omega)]$, but also influences its bandwidth. In Supplementary Figures 9**a** and 9**b**, we show the experimental observations of

$\mathrm{Re}[j/(i\omega)]$ (**a**) and $\mathrm{Im}[j/(i\omega)]$ (**b**) versus the frequency f, which are consistent with the theoretical results (Supplementary Figures 3**c** and 3**d**).

Supplementary Figures 9**c** and 9**d** present $\mathrm{Re}[j/(i\omega)]$ and $\mathrm{Im}[j/(i\omega)]$ as functions of the eigenstate's position expectation values $\langle y\rangle$ and $\langle x\rangle$ for the different frequencies, respectively. We can find that the frequency indeed introduces a shift constant in the linearity between $\mathrm{Re}[j/(i\omega)]$ and y_0 (Supplementary Figure 9**c**). On the contrary, it can tune the slope of the linearity between $\mathrm{Im}[j/(i\omega)]$ and x_0 (Supplementary Figure 9**d**). These results agree with the theoretical ones (Supplementary Figures 4**c** and 4**d**).

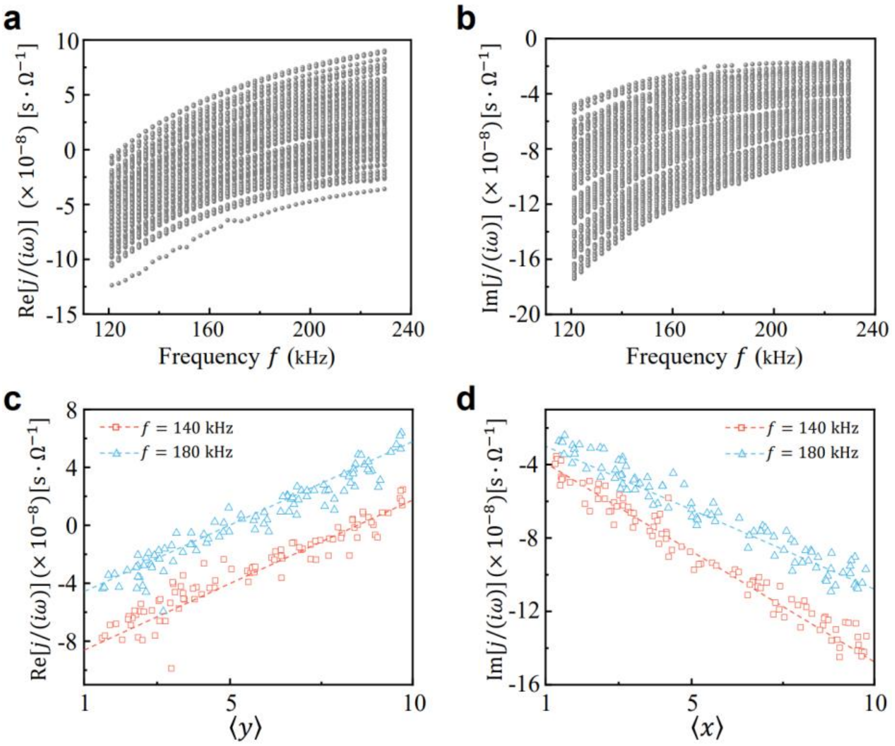

Supplementary Figure 9. **Experimental results of the frequency-controlled admittance spectrum and eigenstates.** (**a**, **b**) Experimental observations of $\mathrm{Re}[j/(i\omega)]$ (**a**) and $\mathrm{Im}[j/(i\omega)]$ (**b**) versus the frequency f. **c** Experimental observations of $\mathrm{Re}[j/(i\omega)]$ versus the eigenstate's position expectation value $\langle y\rangle$ for the different frequencies $f = 140$ and 180 kHz. **d** Experimental observations of $\mathrm{Im}[j/(i\omega)]$ versus the eigenstate's position expectation value $\langle x\rangle$ for the different frequencies $f = 140$ and 180 kHz. The dashed lines in **c** and **d** denote the corrected central trend lines. The parameters are the same as those in Fig.2 of the main text.

Supplementary Note 8: Continuum Landau modes in one-dimensional circuit lattices

As demonstrated in Supplementary Note 2, the pseudomagnetic field induced Landau level modes are localized in 2D space under the symmetric gauge. However, these modes are extended along one dimension if one chooses Landau gauge. This suggests that the CLMs can also be realized in 1D system. In this section, we demonstrate the existence of CLMs in two 1D circuit lattices, which separately corresponds to the vertical and horizontal lattices in Supplementary Figure 1.

We first consider the 1D lattice with non-reciprocal hopping $\pm t_y$ and linear real on-site potential nB_y, whose circuit structure is shown in Supplementary Figure 10**a**. The corresponding circuit Laplacian can be expressed as

$$J(\omega)/i\omega = \sum_n \left[t_y(|n\rangle\langle n+1| - \mathrm{H.c.}) + (nB_y + \epsilon_0)|n\rangle\langle n|\right], \quad (15)$$

where $\epsilon_0 = -i/(\omega R_0) - 1/(\omega^2 L_0)$. It can be written as

$$J_{\mathbf{k}}/(i\omega) = i2t_y \sin k_y + \nu_{\mathbf{k}}\partial_y + B_y y \quad (16)$$

in the continuum limit via the same procedures in Supplementary Note 2. We can find that Supplementary Equation 16 is akin to the non-Hermitian Dirac Hamiltonian under the Landau gauge $\mathbf{A} = (-By, 0)$. The eigenstates are obtained as $\psi(y) = C\exp[\eta(y - y_0)^2/2]$, where $\eta = B_y/\nu_{\mathbf{k}}$ and $y_0 = \mathrm{Re}[j/(i\omega)]/B_y$. These states are CLMs for $\eta < 0$ whose center position are linearly related to the real part of the eigenvalues. In Supplementary Figure 10**b**, we plot the complex admittance eigenvalues together with their corresponding position expectation value $\langle x\rangle$ of the Laplacian matrix (Supplementary Equation 15) for an a.c. frequency $f = 162$ kHz. It is clear that the center positions of the CLMs are linearly dependent with $\mathrm{Re}[j/(i\omega)]$ as expected. This property can be used as "rainbow traps" where the eigenstates are localized at positions proportional to frequency. To clarify this effect, we simulate the steady-state voltage response of the circuit by exciting all nodes, as shown in Supplementary Figure 10**c**. Obviously, the response has a sharp amplitude peak positioned proportional to the excitation frequency.

Another type of 1D circuit lattice featuring the CLMs is sown in Supplementary Figure 11**a**, whose circuit Laplacian is given by

$$J(\omega)/(i\omega) = \sum_m [t_x(|m\rangle\langle m+1| + \mathrm{H.c.}) + (\epsilon_0 - imB_x)|m\rangle\langle m|], \quad (17)$$

where $\epsilon_0 = C_0 - 1/(\omega^2 L_0)$. In the continuum limit, it becomes

$$J_{\mathbf{k}}/(i\omega) = 2t_x \cos k_x + i\mu_{\mathbf{k}}\partial_x - iB_x x. \quad (18)$$

Supplementary Equation 18 amounts to the non-Hermitian Dirac Hamiltonian under the Landau gauge $\mathbf{A} = (0, Bx)$. The corresponding eigenstates are $\psi(x) = C\exp[\eta(x - x_0)^2/2]$ with $\eta = B_x/\mu_{\mathbf{k}}$ and $x_0 = \mathrm{Im}[j/(i\omega)]/B_x$. In this case, the center position of the CLMs is proportional to $\mathrm{Im}[j/(i\omega)]$, which is conformed in Supplementary Figure 11**b**. Since the modes with lowest relative loss occupy a boundary, the steady-state voltage response is concentrated at this boundary acting as a wave funnel, as shown in Supplementary Figure 11**c**.

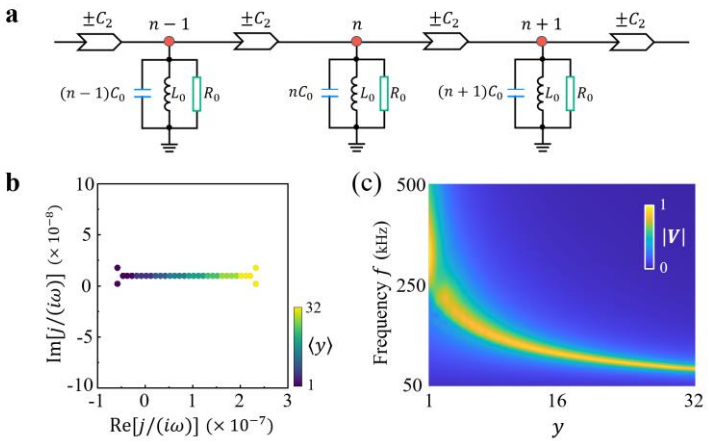

Supplementary Figure 10. **Continuum Landau modes in 1D non-Hermitian electric circuits. a** Schematic diagram of the designed 1D circuit with non-reciprocal hopping and linear real on-site potential. **b** Complex admittance for a.c. frequency $f = 162$ kHz. The color of each point denotes the eigenstate's position expectation value $\langle x \rangle$. **c** Node-dependent voltage amplitudes $|\boldsymbol{V}|$ under steady state excitation at frequency f. The simulating parameters are chosen as $C_0 = 10$ nF, $L_0 = 12.4$ μH, $R_0 = 100\ \Omega$, and $C_2 = 10$ nF.

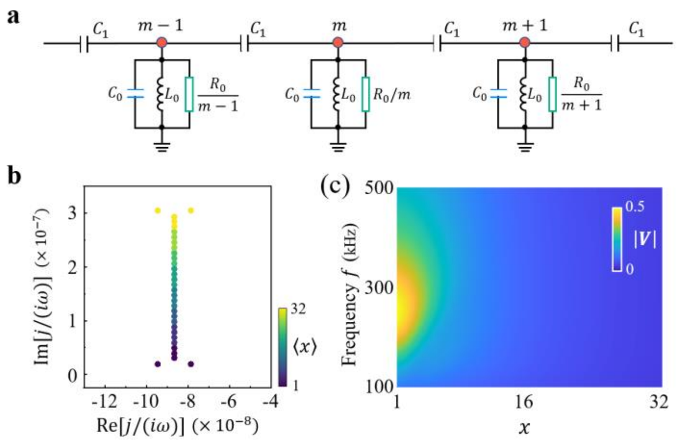

Supplementary Figure 11. **Continuum Landau modes in 1D non-Hermitian electric**

circuits. a Schematic diagram of the designed 1D circuit with reciprocal hopping and linear imaginary on-site potential. **b** Complex admittance for a.c. frequency $f = 162$ kHz. The color of each point denotes the eigenstate's position expectation value $\langle x \rangle$. **c** Node-dependent voltage amplitudes $|\boldsymbol{V}|$ under steady state excitation at frequency f. The simulating parameters are chosen as $C_0 = 100$ nF, $L_0 = 12.4$ μH, $R_0 = 100\ \Omega$, and $C_1 = 10$ nF.

Note that the distributions of the admittance spectra (line or finite area) of our 1D circuit lattices depend on the magnitude of the pseudomagnetic field (i.e., C_0 or R_0). In Supplementary Figures 12**a-c**, we plot the admittance spectrum of the 1D circuit lattice in Supplementary Figure 10**a** for $C_0 = 1$ pF (**a**), $C_0 = 50$ pF (**b**), and $C_0 = 10$ nF (**c**), respectively. One can see that when $C_0 = 50$ pF, the admittance spectrum can fill the complex plane (Supplementary Figure 12**b**), similar to Fig. 3(b) of Ref. (37), and it becomes a line for $C_0 = 1$ pF (Supplementary Figure 12**a**) or $C_0 = 10$ nF (Supplementary Figure 12**c**). The similar distributions of the admittance spectra can also be found in the circuit lattice in Supplementary Figure 11**a** for different $R_0 = 350\ \Omega$ (Supplementary Figure 12**d**), $R_0 = 10$ kΩ (Supplementary Figure 12**e**), and $R_0 = 100$ kΩ (Supplementary Figure 12**f**). In our experiments, we choose the parameters $C_0 = 10$ nF and $R_0 = 100\ \Omega$, in which a linear distribution of the admittance spectrum in the complex plane is considered. It should be emphasized that this linear distribution does not affect the key feature of the CLMs, i.e., the CLMs' center position varies linearly with the complex admittance eigenvalues.

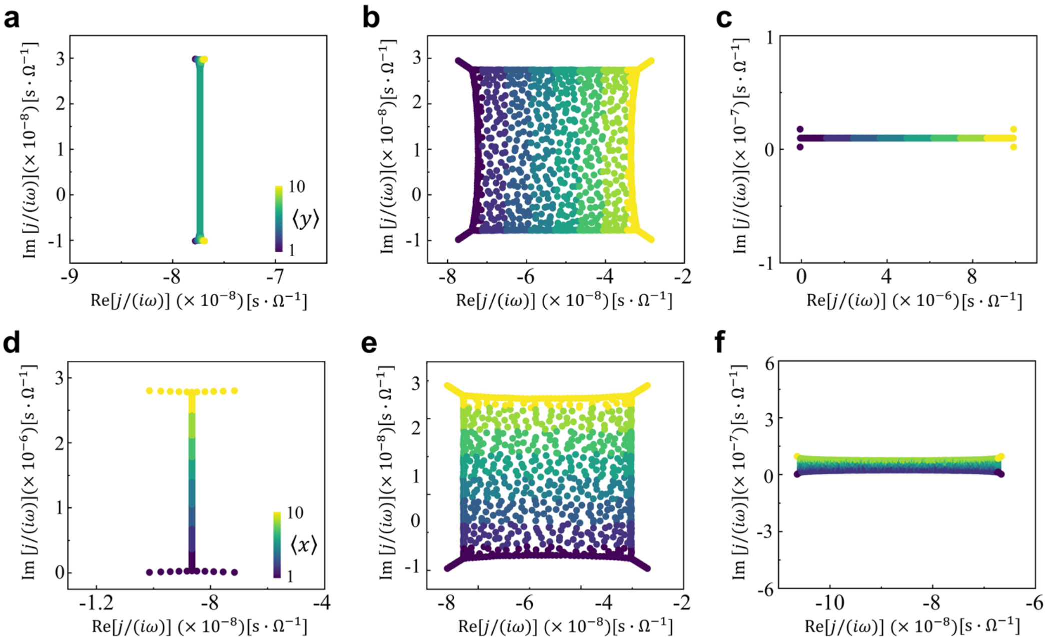

Supplementary Figure 12. **The admittance spectra for different parameter values.** **a-c** Admittance spectra of the circuit Laplacian in Supplementary Figure 4**a** for $C_0 = 1\ \mathrm{pF}$ (**a**), $C_0 = 50\ \mathrm{pF}$ (**b**), and $C_0 = 10\ \mathrm{nF}$ (**c**). The color of each point denotes the eigenstate's position expectation value $\langle y \rangle$. **d-f** Admittance spectra of the circuit Laplacian in Supplementary Figure 4**d** for $R_0 = 350\ \Omega$ (**d**), $R_0 = 10\ \mathrm{k}\Omega$ (**e**), and $R_0 = 100\ \mathrm{k}\Omega$ (**f**). The color of each point denotes the eigenstate's position expectation value $\langle x \rangle$. In all subfigures, the size of the circuit lattice is chosen as 1000. The other parameters are the same as those in Supplementary Figure 4 of the revised manuscript.
